# Creating personas for exposome research: the experience from the HEAP project

**DOI:** 10.12688/openreseurope.15474.1

**Published:** 2023-02-07

**Authors:** Heather Coombs, Tracy Wootton, Joakim Dillner, Heimo Müller, Anouk Berger, Zisis Kozlakidis

**Affiliations:** 1 Learning and Capacity Building, International Agency for Research on Cancer, Lyon, 69337, France; 2 Laboratory Services and Biobanking, International Agency for Research on Cancer, Lyon, 69337, France; 3 Infectious Disease Epidemiology, Karolinska Institute, Stockholm, Sweden; 4 Information Science and Machine Learning, Medical University of Graz, Graz, Austria

**Keywords:** exposome; personas; scientific engagement; environmental exposure; epidemiology

## Abstract

The exposome is a complex scientific field that has enjoyed consistent growth over the last two decades, defined as the composite of every exposure to which an individual is subjected from conception to death. The study of the exposome requires consideration of both the nature of those exposures and their changes over time, and as such necessitates high quality data and software solutions. As the exposome is both a broad and a recent concept, it is challenging to define or to introduce in a structured way. Thus, an approach to assist with clear definitions and a structured framework is needed for the wider scientific and public communication.

Results: A set of 14 personas were developed through three focus groups and a series of 14 semi-structured interviews. The focus groups defined the broad themes specific to exposome research, while the sub-themes emerged to saturation via the interviews process. Personas are imaginary individuals that represent segments/groups of real people within a population. Within the context of the HEAP project, the created personas represented both exposome data generators and users.

Conclusion: Personas have been implemented successfully in computer science, improving the understanding of human-computer interaction. The creation of personas specific to exposome research adds a useful tool supporting education and outreach activities for a complex scientific field.

## Introduction

The rapid advance of technology means that complex scientific issues become an inevitable part of modern research and society in general. A basic understanding of these complex issues is needed if such technological advancement is to gain wide adoption and eventual societal implementation. One of the essential assumptions pursuant to the greater understanding of scientific outcomes is that greater access to information will eventually lead to more knowledge, moving beyond the technical systems where that knowledge has originated from, and becoming a wider scientific and public commodity. This is one of the main pillars supporting the ‘Open Science’ principles as articulated by the European Commission over the last decade ^1– 4^. At the same time, new concepts of the “understanding of science” have emerged, emphasising the needs for “better science communication” directly relating to many fields where past technological advancement has been rapid, for example nanotechnology, molecular biology and -omics technologies, such as genomics ^5– 7^. These needs are expressed equally as strongly whether they relate to narrow-focus applications of new technologies (e.g., molecular genetic tests) ^8^, fields of activity (e.g., infectious diseases) ^9^ or even entire facilities and research infrastructures (e.g., biobanks) ^10– 12^.

One such complex scientific field that has enjoyed consistent growth over the last two decades is the exposome, which is defined as the composite of every exposure to which an individual is subjected from conception to death. Study of the exposome requires consideration of both the nature of those exposures and their changes over time ^13^. The concept was originally developed by Dr Chris Wild as a means of drawing attention to the critical need for more complete environmental exposure assessment in epidemiological studies (‘environmental’ defined in this context in the broad sense of non-genetic). The exposome, therefore, complements the genomic technologies by providing a comprehensive description of lifelong exposure history; and is linked to epidemiological tools so that any outcomes can be utilized for better delineating the causes and prevention of human disease ^14^. However, the exposome is a very broad as well as a recent concept and that makes it challenging to define or to introduce in a structured way. Therefore, an approach to assist with both clear definitions and a structured framework is needed for the wider scientific and public communication, driven through specific implementation cases ^15, 16^. A similar experience was acquired through the B3 Africa project, where the engagement of clinicians and policy makers to biobanking was achieved through well delineated definitions and communication activities ^17^.

To facilitate engagement with the scientific community and to gain a deeper insight for the exposome-derived set of tools, and to establish a clearer context and structure for what the “exposome” means in practice, a series of personas was created within the Human Exposome Assessment Platform (HEAP) project ^18^. Personas are imaginary individuals of any gender that represent segments/groups of real people within a population ^19^. The population represented by such a persona can be specific groups of users of content, a tool or a wider system. Personas have been implemented successfully in computer science, improving the understanding of human-computer interaction often through use cases ^20^, as well as in direct-to-consumer marketing case studies ^21^. Hence, the utilization of personas in the exposome field was a logical extension of such previous communication activities.

The HEAP project is a five-year project funded by the European Union (EU) Horizon 2020 Research and Innovation programme. It aims to provide an informatics platform, populated with research data from cohort studies, national registries, wearable sensors and consumer receipts. The ultimate goal is to make pseudonymized data from large-scale population cohorts; including data on biological samples, from health registries, and from research; safely interoperable and reusable.

A longer-term aim is to create a legacy for HEAP as an exposome research resource by providing training for the wider scientific community, and to clearly communicate the benefits of using HEAP to the target audiences. To achieve the latter effectively and lay the grounds for future adoption, a training and communication strategy must be developed to provide enhanced insights of the process to current and future end-users. This manuscript describes the creation and implementation of end-user personas, specific to the context of the current project, but with the intention that this communication approach can be adapted for wider use in the exposome research field in the future.

## Methods

The two main data sources for the current work were a series of virtual focus group meetings and a number of virtual, individual, semi-structured interviews. More specifically, the personas were generated from collected qualitative data as follows:

i) Information was gathered from three meetings of a small focus group with five participants, during which the initial identification of stakeholder groups and their characteristics were defined. These Persona classifications enabled the selection of the common questions that formed the basis of the subsequent interviews. The virtual focus group participants included experienced professionals with more than five years of experience.
ii) Semi-structured interviews then took place with individuals working on the project during three days of a HEAP workshop. Focus group participants were not involved in the face-to-face interviews, except for one individual due to the specificity of their scientific expertise. The sample size (n=14) of the individuals interviewed in this step is similar to other qualitative studies utilizing virtual focus groups for data collection ^22^. The participants were aged from 20 to 60 years old and a slight majority identified as male (eight out of 14). When asked about their professional designation there were several titles provided; the final professional titles used for the creation of the personas were mutually agreed during the interview. All participants had some prior knowledge of the exposome concept and of the HEAP proposal, though not necessarily exhaustive.

The following questions were asked to all interview participants:

1 “Which HEAP audience group (i.e., “Persona”) does your profile best correspond with?” *(The aim being to match the interviewee to the most appropriate HEAP Audience category as set out in the HEAP proposal. HEAP audience categories are grouped under four main stakeholder categories – Research, Policy maker, Industry, Public: e.g.: “Epidemiologist” (within the “Research” Stakeholder group), or “Citizen” (within the “Public” Stakeholder group))*

2. “What is your profession *?” (Profession: In practice, this served as a sub-category for the above-mentioned Persona/audience group) e.g.: “Medical geneticist” within the “Epidemiologist” Persona*

3. “How do you expect to interact with the HEAP informatics platform?” *(This served to define the Learning Needs Assessment category: e.g., Data provider/data processor/end user, with the aim of later developing targeted learning interventions)*

4. “Which age range are you in?” *(Age ranges were presented in 10-year groups: e.g., 40-50)* The rationale behind asking about age was to reflect the duration of professional experience and level of seniority in the participants’ professional field. 

5. “Tell us a bit more about yourself and your professional background.” This information was listed under the heading “Personal information” and was intended to provide further context and depth to the persona. For example, it allowed us to record that, prior to becoming an epidemiologist, one of our interviewees completed medical school and qualified as a doctor. Another interviewee, participating as a persona in the “Public” stakeholder category, shared details about children and family life as well as educational background.

6. Interests: “What are your motivations for using the HEAP informatics platform or the scientific insights emerging from the HEAP informatics platform?”

7. Ethical considerations: "From your point of view, both professionally and personally, what are the ethical/legal questions or themes around the use of the HEAP platform?” (Prompts included: providing data for analysis, data processing, consent, etc.)

8. Powers/expertise: “What purposes are you able to use the HEAP platform for, and what skills or knowledge do you have, what access to resources/data to achieve this, etc.”

9. Needs: “What do you need in terms of knowledge, resources, access rights etc., in order to make full use of or achieve your aims in regard to the HEAP platform?”

The interviews used an inductive, bottom-up approach for the analysis, which meant making use of meaning and themes formed and verbalized by the participants ^23^. This method was considered more appropriate for the creation of believable and empathic personas. The data were grouped under appropriately relevant themes. These were then presented to the second author, a leading expert at the same faculty, who audited the categories and titles of the themes to ensure their accuracy and depth of specificity. A further member check with the focus group participants also contributed to the trustworthiness of the findings. This research was conducted under IARC ethical approval No.22–37; the anonymous participation in the interview was considered as an indication of consent. The raw data on the personas interviews are available at the Open Science Framework, https://doi.org/10.17605/OSF.IO/VXM3Z.

## Results

The stakeholder groups identified through the initial focus group sessions are listed in Table 1. These were based on the current broad end-user groups of the scientific information of exposome research, and aligned with the categories outlined in the HEAP proposal.

**Table 1.** Stakeholder groups linked to the personal mapping from the focus groups.

Stakeholder group	Stakeholder mapping/personas
Research	Data providers, epidemiologists, biologist/medical researchers, bioinformaticians, biobank managers, Information Communications Technology (ICT) scientists, ethical experts, Applied Artificial Intelligence users
Policy makers	National food agencies, national/international environmental protection agencies, national and international public health agencies, health organizations targeting cancer, health organizations targeting maternity
Industry	Pharmaceutical, food producers, ICT & electronics (Internet of Things (IoT), wearable devices, mobile devices)
Public	Patient organizations, civil society organizations and citizens

Four themes were expected to emerge from the experiential accounts of the virtual interviewees. Each of these will be described along with the subthemes that were identified through the analysis process. The four themes were: (a) the scientific interests in general and of exposome research, (b) the scientific expertise deployed in the use of the exposome platform, (c) the ethical considerations in conducting such research activities and, (d) the needs/wants from both the HEAP project and exposome research in general. Each of these included subthemes, these are shown in Table 2.

**Table 2.** Themes and sub-themes collected from the interviewees on exposome research.

Theme	Sub-theme
Scientific interests	Scientific discovery and impact on health and well-being. Data analyses, especially of large datasets. Creating and using bioinformatics’ pipelines. Understanding the potential and limitations of exposome research. Linking exposome research to precision medicine. Data modelling for diseases. Integrating various software tools
Scientific expertise	Medical doctor; can define clinically relevant questions. Epidemiologist; can understand large-scale population data. Data analyst; can allow dataset interoperability. Public health specialist; can use registry data. Medical researcher; can understand biological functions. Biostatistician; impacts health policy through prediction models. Computer scientist; can code software tools. Citizen scientist; linking to prevention and personal health.
Ethical and Legal considerations	Limitations of working with real-world data (e.g., access to data, ownership of data). Implementation of data-related legal frameworks in various locations (e.g., national context). Consent for the secondary use of existing data. Data location (e.g., physical location of servers vs. open science cloud). Potential Identifiability through the use of data.
Needs or wants from exposome research	New insights from existing datasets (e.g., clinical and/or consumer related). Demonstrate the impact of exposome research in practice. Use existing HEAP pipelines as well as new analytical methods as a result of exposome research. New insights on transferring, collating and analysing data sets. Availability of educational material for exposome research. Simple and efficient interface for digital research tools. Allowing datasets to be Artificial Intelligence (AI) and Machine Learning (ML) ready. Understanding the role and position of the individual citizen.

**Figure 1.**
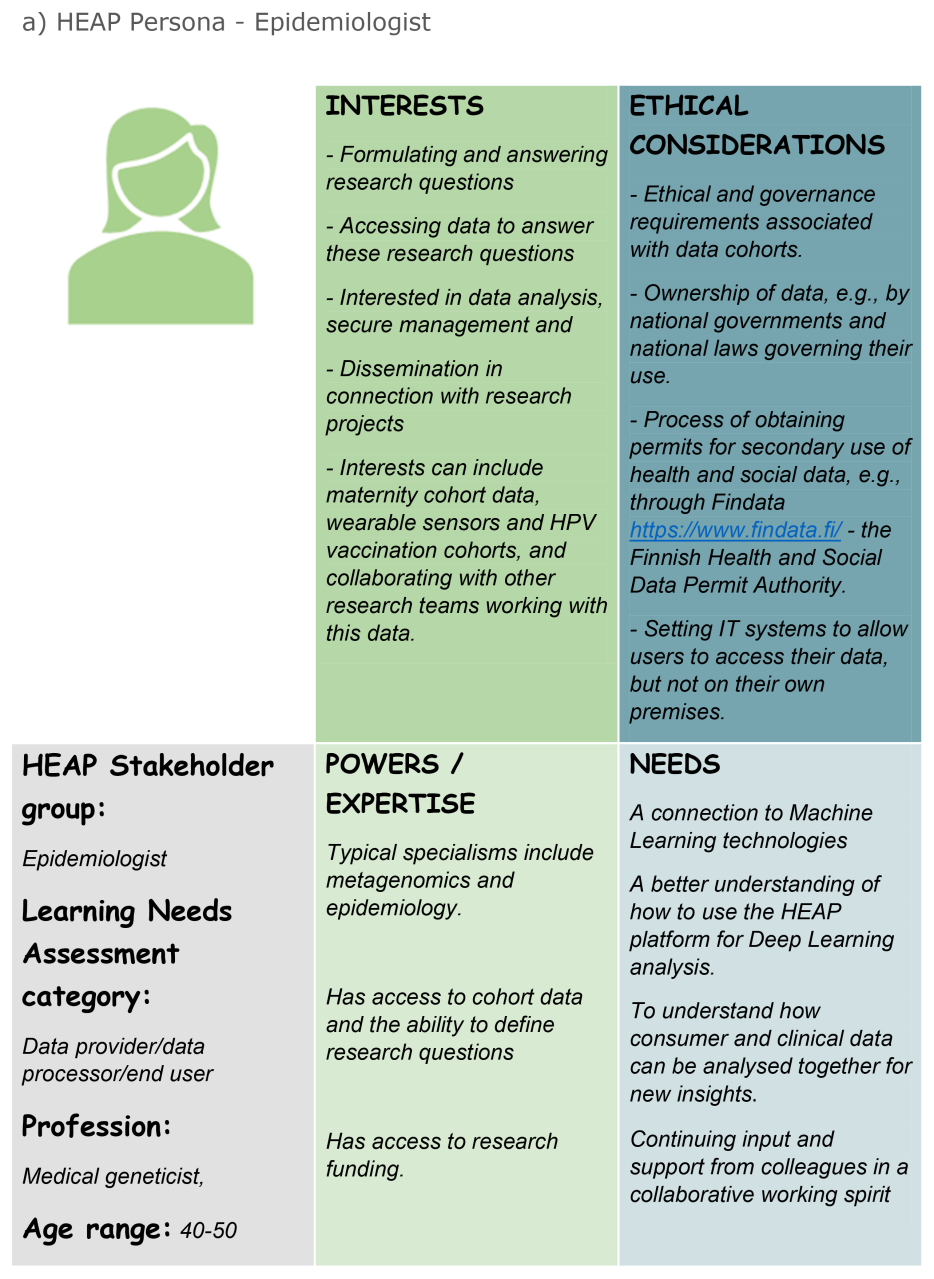
Two of the 14 personas created regarding exposome research: **a**) the Epidemiologist/Medical Geneticist, and **b**) the Public/Citizen.

Two such personas are shown in Figure 1 below.

## Discussion

The themes identified represent broad categories reflective of scientific endeavour in general. As such the underlying commonality of the personas make them potentially transferable to other scientific fields and/or technological implementations. While there are general sub-themes on the impact of research on health and well-being, the creation, use and integration of data tools features strongly throughout all sub-themes. This is expected due to the background of many of the HEAP partners, as well as the emergence of this scientific interest in general. The innovations in healthcare, diagnostics, sensors, and data analysis with advanced methods offer opportunities for improved personalized healthcare, lower costs and benefits to the medical industry ^24^. However, due to the rapid development of technology, the implementation and integration of such tools and platforms is heterogeneous, leading to calls for their greater understanding, and creation of a better-defined process for such utilizations ^25– 27^. This need is more pronounced in the case of a relatively recent concept such as the exposome ^28– 31^. Importantly, the sub-theme of understanding the limitations of exposome research has also emerged, in line with previously published work ^32, 33^.

As the goal of exposome research is to better study the complexity of healthcare realities, it inevitably requires a close connection with many disciplines, such as epidemiology, data analysis and bioinformatics, as is the case in the personas. Having said that, the handling of large and diverse datasets, generated by these different disciplines, raises an entire group of questions relating to the ethical and legal aspects of their use ^34, 35^. While there does not seem to be a consensus approach in responding to those challenges, there are several high-profile projects that have addressed them within the context of their specific research aims ^36, 37^. The last set of sub-themes relate to the forward-looking application of the exposome, understanding the ability to provide further insights, to apply advanced analytical tools (such as AI and ML), while doing so in a manner that will be ‘simplified’, so that wider adoption can be enhanced.

Moreover, two separate sub-themes emerged: educational support towards understanding the exposome and the tools applied therein; and the role of the citizen scientist. The educational need is self-evident, as for any scientific or technological innovation and can range from the educational needs of students ^38^ to those of professionals ^39, 40^. On a separate note, the role of the citizen scientist has been strongly encouraged by the European Commission, both in strategic statements ^41^ and in practice by the creation of the open science environments on the cloud ^42, 43^. As such it is not a surprise that this parameter would emerge for an EU-based project, however it also highlights the underlying educational need for the wider public.

The personas created as part of HEAP are designed as a gender-independent communications and training tool to enable the consortium participants to understand experiences and backgrounds that differ from their own. This is particularly important for cross-disciplinary teams (as there are on most exposome studies) that may benefit in the long-term, as personas may represent a unifying compass for goal alignment ^44^. In the context of the HEAP project, the information gathered in the form of personas was used by the topic experts (in ethical/legal, technical, data management, and bioinformatics) to develop Learning Needs Assessments for future users of the HEAP platform. The personas enabled the topic experts to identify the strengths, knowledge/skills gaps and motivations of the HEAP target audiences through an engaging presentation of their “Needs”, and their “Interests”, “Ethical Considerations” and “Powers/expertise”.

However, there are certain limitations to the current work. Firstly, the data were manually gathered and then manually analysed to develop the personas, resulting in a relatively low number produced, and a low rate for potential further customization. Furthermore, the personas are not necessarily representative of all the types of stakeholders originally identified. Having said that, this number is sufficient for the needs of the HEAP project, and the methodology can be replicated so that further personas are produced from a larger data set in the future. A further limitation is the impact of this work through established communication channels. While the HEAP project is active on several social platforms, these are not exhaustive, and as such the use of the personas at present would remain targeted.

Having reflected on the questions asked during the Personas interviews, the following lessons learnt were identified and will be applied to future iterations of the questionnaire:

- Age range: This question will be adapted to focus not on age, but on years of professional experience and seniority. This is because age is not always an accurate reflection of years of experience in a professional field, due to the increasing tendency towards mid-life career changes, such as starting a research degree in later life.
- Gender: To ensure that the HEAP platform is designed to meet the needs and expectations of all genders, a question about gender identity will be included in the future. This might enhance identifiability of users with the developed persona.

As the HEAP informatics platform nears completion, the focus will move beyond the academic research community to the “Personas” who will be concerned with the research insights and outputs of HEAP, such as Policy Makers and the Public. Future persona interviews will be conducted with these stakeholder groups to tailor dissemination materials and the project communication strategy to maximise the impact of the project. The end result will provide a comprehensive overview, using Personas, of all the main stakeholder groups of the project.

## Conclusion

The rapid technological advancement has been able to support new scientific concepts, such as the exposome. However, in doing so the scale of data required and complexity has also increased, often exponentially. It is anticipated that a greater understanding of complex issues will in turn lead to enhanced ability on the part of individuals and communities to deal with these issues when they encounter them. To achieve the latter appropriate tools are required, and the creation of personas is one such tool. Here we describe the creation of personas for the EU-funded exposome project HEAP. The conceptual capabilities of the personas as an interface to analytical systems present considerable promise, both for enhancing the understanding of the methods themselves, as well as for data-driven interpretations within healthcare. Indeed, they can potentially offer considerable impact for researchers and organizations that desire to understand their end-user needs and vice versa.

## Data Availability

Open Science Framework. Human Exposome Assessment Platform. DOI: https://doi.org/10.17605/OSF.IO/VXM3Z This project contains the following data: This is a new registration. It contains the raw data files for the educational aspects of HEAP. Data are available under the terms of the Creative Commons Zero "No rights reserved" data waiver (CC0 1.0 Public domain dedication).
