# Supplementary material for: Creating personas for exposome research: the experience from the HEAP project
Source: Open Res Eur. 2023 Feb 7;3:28. [Version 1] doi: 10.12688/openreseurope.15474.1 (PMC10445937; doi:10.12688/openreseurope.15474.1)
Supplement: Peer review history

# Peer review: Reviewer response for version 1

**DOI:** 10.21956/openreseurope.16730.r32254

**Published:** 2023-06-16

**Authors:** Yuan Yao

**Affiliations:** 1 Department of Environmental Health Sciences, University of California Los Angeles, Los Angeles, CA, USA; 2 College of Environmental Sciences and Engineering, Peking University, Beijing, Beijing, China

The article explores the use of personas in exposome research, which focuses on understanding how long-term environmental exposures impact human health. The study created 14 personas through focus groups and interviews to represent different user groups in this field. These personas play a crucial role in educational and outreach activities by enhancing communication and understanding of the complex exposome concept. 

 The article highlights the importance of clear definitions and structured frameworks in scientific and public discussions related to exposome research. In addition, the personas developed in this study can be applied to other scientific fields and technological implementations, promoting better collaboration and understanding.

 The current manuscript is well written, but there are some flaws in statistical analysis and data interpretation. Some additional rationale of the approach taken, and potentially additional analyses, may be warranted.

Is the study design appropriate and does the work have academic merit?

Yes

Is the work clearly and accurately presented and does it cite the current literature?

Yes

If applicable, is the statistical analysis and its interpretation appropriate?

Partly

Are all the source data underlying the results available to ensure full reproducibility?

Yes

Are the conclusions drawn adequately supported by the results?

Yes

Are sufficient details of methods and analysis provided to allow replication by others?

Yes

Reviewer Expertise:

Environmental exposure assessments, biological mechanisms underlying air pollution-related health issues, sub-population susceptibility to pollution exposure, and the nexus of air pollution, climate change, and human health.

I confirm that I have read this submission and believe that I have an appropriate level of expertise to confirm that it is of an acceptable scientific standard.

# Peer review: Reviewer response for version 1

**DOI:** 10.21956/openreseurope.16730.r30826

**Published:** 2023-02-22

**Authors:** Eleni Fthenou

**Affiliations:** 1 Qatar Biobank for Medical Research, Qatar Foundation for Education, Science, and Community, Doha, Qatar

The article describes the creation of 14 end-user personas, imaginary individuals, gender-independent, representing groups of real people within a population. The overall aim is for the personas to be used as both exposome data generators and users under the context of the HEAP project. Personas will be used as exposome data generators and users in communication and training tools engaging scientific community, more particularly the HEAP consortium. 

 The personas were generated from collected qualitative data from virtual focus groups applying semi-structured interviews. Four themes reflecting general scientific endeavor in exposome research, the ethical aspect of such research and the HEAP needs and requirements were identified. The conceptual capabilities of the end-user personas identified in this study is expected to ease the understanding of individuals and communities to deal with complex issues enhancing the procedures themselves as well as they encounter them in data-driven interpretations.

 The current manuscript presents the work done in a clear and accurate way by providing adequate access to supplementary data and citing current literature. The study design is clearly presented along with its strengths and weakness. The scientific merit is clearly stated as the outcomes identified represent broad categories of scientific endeavor with commonalities of the personas make them potentially transferable to other scientific fields.

Is the study design appropriate and does the work have academic merit?

Yes

Is the work clearly and accurately presented and does it cite the current literature?

Yes

If applicable, is the statistical analysis and its interpretation appropriate?

Not applicable

Are all the source data underlying the results available to ensure full reproducibility?

Yes

Are the conclusions drawn adequately supported by the results?

Yes

Are sufficient details of methods and analysis provided to allow replication by others?

Yes

Reviewer Expertise:

Environmental and molecular epidemiology, Biobanking.

I confirm that I have read this submission and believe that I have an appropriate level of expertise to confirm that it is of an acceptable scientific standard.
